# Metabolic side effects of clozapine in patients at south ceredigion community mental health team

**DOI:** 10.1192/bjo.2021.905

**Published:** 2021-06-18

**Authors:** Harish Reddy

**Affiliations:** Hywel Dda University Health Board

## Abstract

**Aims:**

The aim of the audit was to identify patients at risk of developing Metabolic Syndrome who are on Clozapine in the community. Anyone who has three of following attributes has Metabolic Syndrome. A large waist size (greater than 40 inches in men or 35 inches in women) ,high blood pressure (130/85 mm Hg or higher) ,high triglycerides — a form of fat in the blood (150 mg/dL or higher) ,high blood sugar (a fasting level of 100 mg/dL or higher).Patients receiving should be regularly monitored under clinical review particularly in relation to side effects of the drug and maintain minimum standards of review both physically and clinical investigations once a year .

**Background:**

To measure the screening of central obesity, Blood Pressure, serum glucose levels and lipid profile in last one year.

**Method:**

Data were collected from Blood results and electronic entries of patients who are on Clozapine in South Ceredigion Community Mental Team. There were 31 patients of which 20 were male and 11 were female patients. The age range was 31–66 years and average was 46 years.

**Result:**

52% of the patients had obesity,34 % with Hypertension,50 %Dyslipidaemia and 43 % had Increased glucose tolerance. 80 % were only on clozapine,3% were on combined Amisulpride, 10% on combined on Ariprazole, 3 % on combined Quetiapine.

**Conclusion:**

Treatment of causes like making changing lifestyle changes, weigh reduction using health diet and to include regular physical activity. Reduce Abdominal Obesity and in possible provide nutritional intervention.

